# Targeted Reducing of Tauopathy Alleviates Epileptic Seizures and Spatial Memory Impairment in an Optogenetically Inducible Mouse Model of Epilepsy

**DOI:** 10.3389/fcell.2020.633725

**Published:** 2021-02-18

**Authors:** Yang Gao, Jie Zheng, Tao Jiang, Guilin Pi, Fei Sun, Rui Xiong, Weijin Wang, Dongqin Wu, Shihong Li, Huiyang Lei, Huiling Yu, Qiuzhi Zhou, Ying Yang, Huaqiu Zhang, Jian-Zhi Wang

**Affiliations:** ^1^Department of Pathophysiology, Key Laboratory of Ministry of Education for Neurological Disorders, School of Basic Medicine, Tongji Medical College, Huazhong University of Science and Technology, Wuhan, China; ^2^Department of Pharmacology, Key Laboratory of Basic Pharmacology of Ministry of Education and Joint International Research Laboratory of Ethnomedicine of Ministry of Education, Zunyi Medical University, Zunyi, China; ^3^Department of Neurosurgery, Key Laboratory of Ministry of Education for Neurological Disorders, Tongji Hospital, Tongji Medical College, Huazhong University of Science and Technology, Wuhan, China; ^4^Co-innovation Center of Neuroregeneration, Nantong University, Nantong, China

**Keywords:** epilepsy, cognitive impairment, tau hyperphosphorylation, optogenetics, mouse model

## Abstract

Intracellular deposition of hyperphosphorylated tau has been reported in the brain of epilepsy patients, but its contribution to epileptic seizures and the association with spatial cognitive functions remain unclear. Here, we found that repeated optogenetic stimulation of the excitatory neurons in ventral hippocampal CA1 subset could induce a controllable epileptic seizure in mice. Simultaneously, the mice showed spatial learning and memory deficits with a prominently elevated total tau and phospho-tau levels in the brain. Importantly, selective facilitating tau degradation by using a novel designed proteolysis-targeting chimera named C4 could effectively ameliorate the epileptic seizures with remarkable restoration of neuronal firing activities and improvement of spatial learning and memory functions. These results confirm that abnormal tau accumulation plays a pivotal role in the epileptic seizures and the epilepsy-associated spatial memory impairments, which provides new molecular target for the therapeutics.

## Introduction

Epilepsy is characterized by spontaneously recurring epileptic seizures, with cognitive decline generally occurring as a major comorbidity (So and Penry, 1981; Breuer et al., 2016; Witt and Helmstaedter, 2017). The degree of the epilepsy-induced cognitive impairments varies, depending on the age at seizure onset; location, frequency, and duration of epilepsy; and the history of antiepileptic medication (Avanzini et al., 2013; Witt and Helmstaedter, 2017; Feldman et al., 2018), with the mechanisms not yet fully understood, to date. As a result, there is currently no targeted therapeutic strategy to prevent or halt the development the cognitive decline in epilepsy patients (Paudel et al., 2019).

Hyperphosphorylation and intracellular accumulation of tau protein dysregulate microtubule assembly and aggravate the formation of neurofibrillary tangles, thus remodeling neuronal synapses and exacerbating cognitive decline in a collection of diseases named tauopathies, including Alzheimer disease (AD; Wang et al., 2014; Wang and Mandelkow, 2016; Yang and Wang, 2018). Recent studies also revealed prominent accumulation of hyperphosphorylated tau in the brain of temporal lobe epilepsy patients (Tai et al., 2016; Smith et al., 2019). Given that epilepsy and early stage AD share many neurological characters and psychiatric symptoms (Kanner, 2009; Cheng et al., 2015; Zarea et al., 2016), we wonder whether tau pathology also contributes to the cognitive deficits in epilepsy. Furthermore, although knockdown or knockout of tau proteins have been evidenced to significantly attenuate neuronal network hyperexcitability in the brain of mice with epilepsy (DeVos et al., 2013; Holth et al., 2013), whether and how the abnormal accumulation of hyperphosphorylated tau dysregulates neuronal firing activities thus to affect epileptic seizures in epilepsy still remain to be elucidated.

The current epileptic animal models, produced by systemic or focal injection of convulsant agents such as kainic acid or pilocarpine, always show more extensive neuronal damages than those observed in the clinical patients, and the animal death rate is high (Morimoto et al., 2004). Recently, optogenetics has been used to induce sporadic seizures with high temporal and spatial specificity in animal studies (Osawa et al., 2013; Khoshkhoo et al., 2017; Cela et al., 2019). In the present study, we found that optogenetic stimulation of vCA1 excitatory neurons could induce a controllable epileptic seizure with spatial memory deficit in mice. Simultaneously, tau hyperphosphorylation and accumulation were shown, while targeted reducing phospho-tau accumulation attenuated optogenetics-induced epileptic seizures with improved spatial learning and memory.

## Results

### Optogenetic Stimulating vCa1 Excitatory Neurons Induces Epileptic Seizures With Impaired Spatial Learning and Memory

**FIGURE 1:**
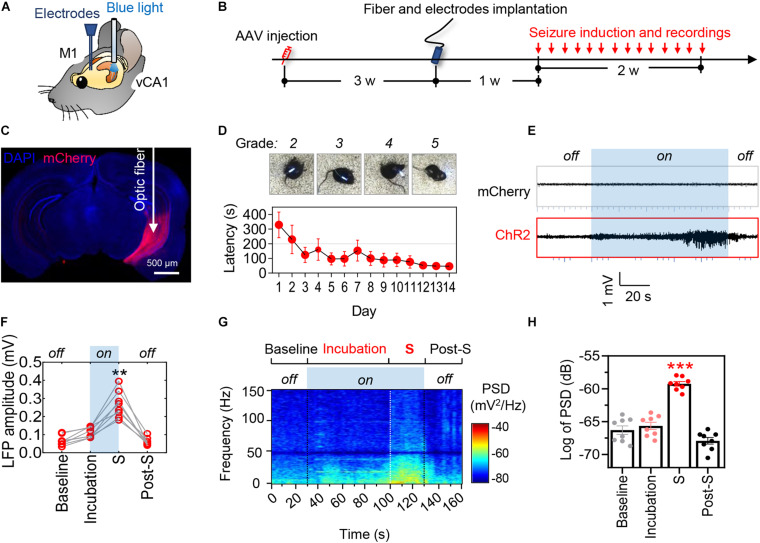
Optogenetic stimulation of ventral hippocampal excitatory neurons induces controllable epileptic seizures. **(A)** Schematic illustrates the locations of optic fiber and recording electrode. **(B)** Experimental procedures of virus injection, optic fiber and electrode implantation, optogenetic seizure induction. **(C)** A representative image showing the AAVs-mediated mCherry expression in the ventral hippocampus. Scale bar, 500 μm. **(D)** The representative images show typical seizure behavior (MRS stages 2–5) and the latency to generalized seizures **(upper)**. Repeated optogenetic stimulation of vCA1 excitatory neurons induced typical seizure behavior, and the latency to generalized seizures was decreasing during repeated induction that showed seizure threshold declined over time (bottom). **(E,F)** Optogenetic hyperactivation of vCA1 excitatory neurons (i.e., blue light on) induced epileptiform neuronal activities in the primary motor cortex (M1). The local field potential (LFP) amplitude in M1 significantly increased during the phase of incubation and generalized seizures (shortened as S). The mice injected with AAV-mCherry were tested as non-seizure controls. Repeat-measures one-way ANOVA followed by Tukey multiple-comparisons tests, **p < 0.01, ***p < 0.001, compared with the baseline, *n* = 8 in each group. **(G,H)** Optogenetic induction of seizures increased the power spectra density (PSD) of LFP in the phase of incubation and generalized seizures. Repeated-measures one-way ANOVA followed by Tukey multiple-comparisons tests. **p < 0.01, ***p < 0.001, compared with the baseline, *n* = 8 in each group. Values are presented as the mean ± SEM.

**FIGURE 2:**
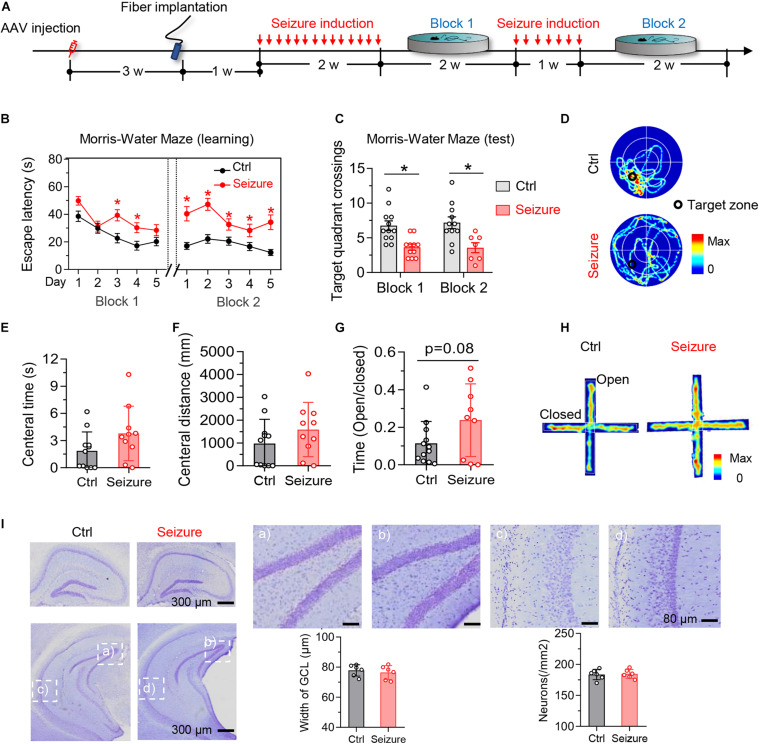
Optogenetic induction of repeated seizures impairs spatial learning and memory without causing anxiety-like behaviors or hippocampal neuron loss in mice. **(A)** Experimental procedures of virus injection, optic fiber implantation, optogenetic seizure induction, and behavioral tests. **(B)** Repeated seizure induction caused spatial learning deficits shown by the increased escape latency during training phase. Repeated-measures ANOVA followed by Tukey multiple-comparisons tests, *p < 0.05. **(C,D)** Repeated seizure induction caused spatial memory deficits shown by the decreased number of target quadrant crossings in water maze test **(B)** and the representative heatmaps **(C)** show traveling time and the trace. Unpaired *t* test, *p < 0.05. **(E,H)** Repeated but controllable seizures did not induce anxiety-like behavior shown by the unchanged central time **(D)** and traveled distance **(E)** in open field test, and the increased open arm stay during elevated plus maze test **(F)**. The representative heatmaps **(G)** show traveling time and the trace. Unpaired *t* test, *p < 0.05. **(I)** Repeated but controllable seizures did not induce neuron loss in the granular cell layer (GCL) of dorsal and ventral hippocampus measured by Nissl staining (*p* > 0.05). Scale bars were as indicated in each panel. *n* = 6 mice in each group. Unpaired *t* tests. Values are presented as the mean ± SEM.

To establish a mouse model of temporal lobe epilepsy with high spatiotemporal controllability of seizures, we stereotaxically injected pAAV-CaMKIIα-ChR2(H134R)-mCherry into unilateral mice ventral hippocampal CA1 (vCA1) (Figures 1A–C). Mice received optogenetic stimulation once each day for 14 consecutive days (block 1) or 21 days (block 2) to mimic sporadic and repeated seizures in clinic (Figure 2A).

Optogenetically hyperactivating vCA1 excitatory neurons with blue light (482 nm, 20 Hz) induced epileptiform discharges in ipsilateral primary motor cortex (M1) (Figure 1D). The amplitude and power spectra density (PSD) of M1 local field potential (LFP) were increased prominently during incubation and ictal periods of epilepsy (Figures 1E–H). Consistently, behavioral seizures of stages 2–6 by modified Racine scale (MRS; Racine, 1975; Haas et al., 1990) were observed (Figure 1D, upper; Video 1). As expected, the epileptic seizures gradually stopped following the cessation of optogenetic stimulation. Further studies revealed that the latency to generalized seizures (MRS stage ≥ 4) decreased with repeated induction of epilepsy over time (Figure 1D, bottom).

By Morris water maze (MWM) test, we observed that repeated seizures for 14 consecutive days (block 1) increased the latency to find the hidden platform at days 3–4 during the training phase (Figure 2B, block 1). Besides, epileptic mice also showed decreased target quadrant crossings at day 6 (Figure 2C, block 1). These data indicate that repeated seizures impair both spatial learning and memory of mice. To examine the effect of longer repeat seizures on spatial learning and memory, we stimulated the mice for another 7 consecutive days (block 2). The additional optogenetic stimulation could also induce epileptic seizures with more serious impairments of spatial learning and memory in MWM test (block 2 in Figures 2B–D).

The optogenetics-induced seizures did not affect the time and distance of mice traveled in the central area in open-field test (Figures 2E,F) nor changed the ratio of open-arm entries in the elevated-plus maze test (Figures 2G,H). These results indicated that the repeated induction of seizures using optogenetics did not induce significant anxiety-like behavior, which was generally reported in typical epileptics models (Baxendale et al., 2005; Inostroza et al., 2012; Scott et al., 2017; Yogarajah and Mula, 2019).

### Epileptic Seizure Induces Phospho-Tau Accumulation Without Hippocampal Neuron Loss in Mice

**FIGURE 3:**
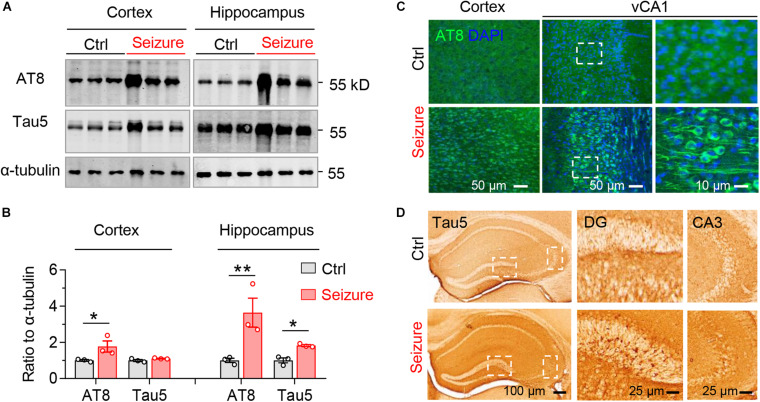
Repeated seizures upregulate phosphorylated and total tau levels in the hippocampus and cortex of mice. Repeated seizures increased phospho-tau (AT8) and total tau (Tau5) levels in hippocampus and cortex measured by Western blotting **(A,B)** and immunostaining **(C,D)**. *n* = 3 mice in each group. Unpaired *t*-tests, *p < 0.05, **p < 0.01. Scale bars were as indicated in each panel. Values are presented as the mean ± SEM.

We next examined the effect of repeated seizures on tau. The levels of tau phosphorylated at the Ser199/Ser202/Thr205 epitope (recognized by the AT8 antibody) significantly elevated both in the cortex and hippocampus of mice after 21 times of repetitive optogenetic induction of seizures (Figures 3A–C). The total tau (recognized by the Tau5 antibody) was also upregulated in the hippocampus of mice with epilepsy (Figures 3A–D).

No significant loss of hippocampal neurons was detected after repeated induction of seizures (Figure 2I), although the neuron death was commonly seen in the drug-induced epilepsy models (Morimoto et al., 2004). These data also suggest that the seizures-induced learning and memory deficits were not caused by neuron death.

### Selectively Facilitating Tau Degradation Using a Proteolysis-Targeting Chimera Effectively Ameliorates Seizures and the Associated Memory Deficits

To directly test the contribution of seizures-induced elevation of phospho-tau and total tau to the seizure inductions and spatial learning and memory impairments, we used a novel small-molecule proteolysis-targeting chimera termed C4 to selectively promote tau degradation (more key information about C4 at https://www.cnipa. gov.cn/with a patent publication number *CN111171113A*).

**FIGURE 4:**
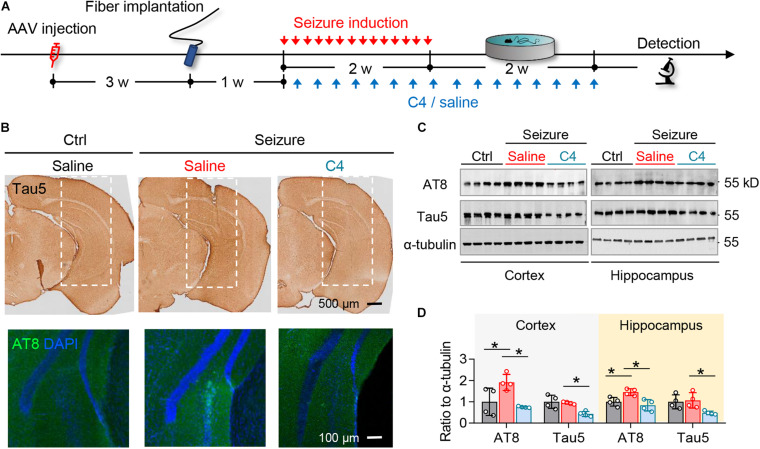
C4 selectively facilitates tau degradation in the optogenetically inducible mouse model of epilepsy. **(A)** Experimental procedure of virus injection, seizure induction, C4 administration, and behavioral tests. **(B–D)** C4 administration diminished the repeated seizure-induced elevation of total tau and phospho-tau levels in hippocampus and cortex measured by immunostaining **(B)** and Western blotting **(C,D)**. *n* = 4 mice in each group. One-way ANOVA followed by Tukey multiple-comparisons tests, *p < 0.05. Scale bars were as indicated in each panel. Values are presented as the mean ± SEM.

**FIGURE 5:**
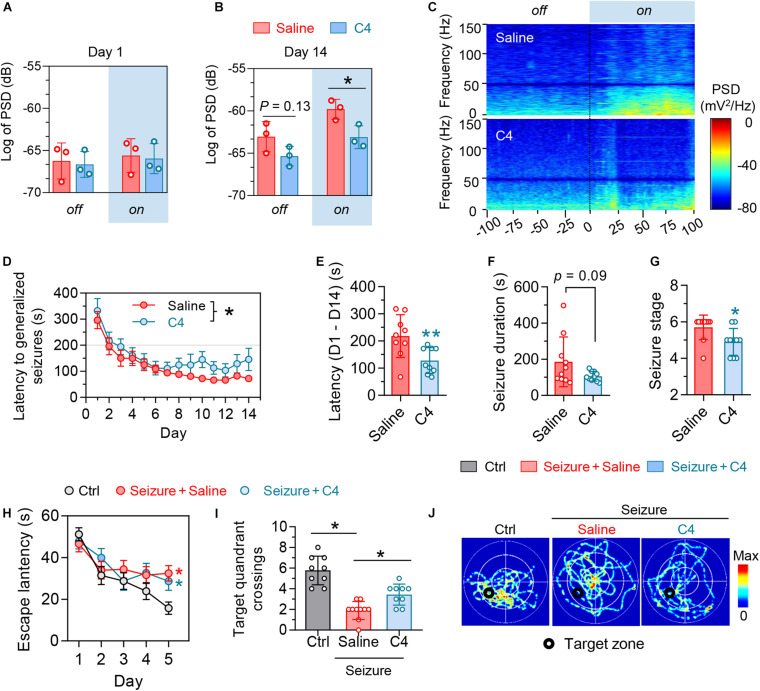
Selectively facilitating tau degradation by C4 alleviates the optogenetics-induced epileptic seizures and the associated spatial memory deficits. **(A–C)** Blue light on did not change basal PSD in primary motor cortex (M1) at day 1, whereas C4 treatment significantly downregulated the PSD in M1 at day 14 during light-on. *n* = 3 mice in each group, unpaired *t* tests, *p < 0.05. **(D,E)** C4 prolonged the latency of generalized seizures of mice. *n* = 7–9 mice in each group. Repeated-measures ANOVA followed by Tukey multiple-comparisons tests, *p < 0.05. Unpaired *t*-tests, **p < 0.01. **(F,G)** C4 tended to decrease the seizure duration and severity of seizure stages at day 14. Unpaired *t* tests, *p < 0.05. **(H–J)** C4 showed limited effect on improving the repeated seizures-induced spatial learning impairments in the water maze training phase. *n* = 9–10 mice in each group. Repeated-measures ANOVA followed by Tukey multiple-comparisons tests, *p < 0.05, compared with the non-seizure Ctrl group. **(I,J)** C4 significantly alleviated the seizures-induced spatial memory impairments. One-way ANOVA followed by Tukey multiple-comparisons tests, *p < 0.05. Values are presented as the mean ± SEM.

Mice received repeated opto-stimulation for 14 days and were subcutaneously administered C4 (3 mg/kg, twice a week for 4 weeks) (Figure 4A), and then the levels of phospho-tau and total were measured. The results showed that C4 efficiently downregulated both phospho-tau (AT8 epitope) and total tau in the cortex and hippocampus of the epileptic mice (Figures 4B–D). Tau reduction by C4 ameliorated the optogenetics-induced seizures as shown by a prolonged latency to generalize seizures, decreased seizure duration, and the severity of seizure compared with vehicle-administered controls (Figures 5A–G). Although C4 showed limited effects in alleviating the seizures-induced spatial learning deficits during the training phase in MWM test (Figure 5H), C4 significantly improved the spatial memory as indicated by the increased target quadrant crossings (Figures 5I,J).

These data together indicate that tau accumulation plays a critical role in repeated seizures and spatial cognitive impairment; targeting tau is promising in alleviating seizures and the associated spatial memory deficits in epilepsy.

## Discussion

Optogenetics provides excellent tools to control neuronal activities with high spatiotemporal specificity (Mondoloni et al., 2019), which gains increasing attention in the study of epilepsy (Choy et al., 2017; Tønnesen and Kokaia, 2017). There are several advantages of these optogenetics-based models compared with classical drug-induced status epileptics models. First, optogenetic tools enable cell type- and location-specific induction of seizures. In the present study, we targeted ventral hippocampal excitatory neurons to mimic the etiology of temporal lobe epilepsy. Second, optogenetics brings higher accuracy for quantitatively controlling the onset, frequency, and severity of seizures. Third, by contrast to classical drug-induced status epileptics models, which generally cause approximately 10–44% animals death (Auladell et al., 2017; Welzel et al., 2020), optogenetics-inducible epilepsy model has higher success rate and repeatability and almost no mortality. In the present study, we established a novel mice model of optogenetically inducible sporadic epilepsy with high spatiotemporal specificity and stable repeatability.

Tau is a microtubule-associated protein mainly engaged in the microtubule stabilization and axonal transport under physiological conditions. Hyperphosphorylation of tau hinders its degradation, thus leading to the aggregation of tau and forming of neurotoxic paired helices (Sadqi et al., 2002). Abnormal accumulation of hyperphosphorylated tau in the hippocampus or appearance of neuropil threads and NFTs in the resected epileptogenic temporal lobe has been reported in a majority of epilepsy patients (Tai et al., 2016; Smith et al., 2019). Consistently, we found that repeated induction of seizures prominently elevated phosphorylated and total tau levels in the cortex and hippocampus. However, whether and how the tau pathology is involved in epilepsy remain not fully understood. Here, using a specific chimera to facilitate tau degradation in an optogenetically inducible mice model of epilepsy, we found a contribution of intraneuronal phospho-tau accumulation to epileptic seizures and associated spatial memory deficits.

However, molecular mechanisms underlying how epilepsy resulted in phospho-tau accumulation still remain to be elucidated. Given that the tau hyperphosphorylation and accumulation generally resulted from upregulated activity of kinase, such as cyclin-dependent kinase 5 (CDK5) and glycogen synthase kinase-3β (GSK-3β), or downregulated protein phosphatase 2A (PP2A) activity (Iqbal et al., 2009)I, the observed increase of phospho-tau in epilepsy might also be attributed to the dysfunction of these kinase or phosphatases. Indeed, increased activity of glutamatergic neurons has been reported to induce tau hyperphosphorylation through PP2A inhibition by releasing of synaptic Zinc in cultured hippocampal neurons and brain slices of AD mice (Sun et al., 2012). Similarly, CDK5 and GSK-3β were both reported to be overactivated in temporal lobe epilepsy (Liu et al., 2017). Whether these proteins change in the optogenetics-induced epilepsy and their direct links to the abnormal tau accumulation still remain to be elucidated.

Another intriguing question is how the intraneuronal accumulation of phospho-tau dysregulates epileptic seizures. The hyperphosphorylation and abnormal aggregation of tau protein might directly affect neural network activities by causing microtubule depolymerization (Verstraelen et al., 2017) or result in excitation/inhibition imbalance by dysregulating the release of neurotransmitters (Holth et al., 2013; Sánchez et al., 2018). However, it seems currently elusive how tau accumulation determines neuronal network activities. Although mice expressing mutated human tau exhibited suppressed neuronal activity (Busche et al., 2019; Sohn et al., 2019), selective overexpressing human tau in GABAergic interneurons resulted in hyperactivation of neighboring excitatory neurons (Zheng et al., 2020). It deserved further investigation how the hyperphosphorylated tau was distributed in different subsets of neurons in epilepsy.

We have previously evidenced that intracellular accumulation of hyperphosphorylated tau dysregulated neuronal plasticity and interneuronal transmissions in AD (Yin et al., 2016; Ye et al., 2020), the most common form of dementia in the elderly. In fact, neuronal hyperactivation and epileptic seizure were commonly reported in the early stage of AD (Lam et al., 2017; Baker et al., 2019). Thus, the pathological tau-induced neuronal dysfunctions, like those in AD, might also at least partly contribute to the cognitive impairment in epilepsy.

Most importantly, we found that specifically reducing tau with a proteolysis-targeting chimera in the brain significantly inhibited epileptic seizures, as well as alleviated spatial memory impairment in our mice model. Consistently, non-selectively facilitating tau dephosphorylation by enhancing the enzyme activity of PP2A with sodium selenite effectively prolonged the time of epilepsy induction, and reduced the number and degree of seizures (Jones et al., 2012; Liu et al., 2016). Moreover, knocking down or out of the endogenous tau also reduced the frequency and severity of seizures in mice (DeVos et al., 2013; Holth et al., 2013; Gheyara et al., 2014).

Taken together, the present study found that optogenetic overactivation of vCA1 excitatory neurons induced epileptic seizures and cognitive impairments, associating with abnormal accumulation of phospho-tau in hippocampal neurons. Further studies revealed that specific facilitating tau removal partly rescued the epileptic seizures and the associated spatial memory deficits. These findings confirm that abnormal tau accumulation plays a pivotal role in optogenetics-induced epileptic seizures, and targeting tau may be promising in treatment of epilepsy.

## Materials and Methods

### Animals

Adult (8–12 weeks) male C57BL/6 mice weighing 20–30 g were purchased from Beijing Vital River Laboratory Animal Technology Co., Ltd. All mice were housed in groups of four to five per cage and were housed under a 12-h light–dark cycle (lights on at 7:00 PM and off at 7:00 AM) at a stable temperature (23–25°C). Food and water were available *ad libitum*. All animal studies had complied with all relevant ethical regulations for the animal testing and research and were approved by institutional guidelines and the Animal Care and Use Committee of Huazhong University of Science and Technology.

### Virus Injection and Optic Fiber Cannula Implantation

Mice were anesthetized with 1% pentobarbital sodium (35 mg/kg) and head-fixed in a stereotactic frame (RWD, Shenzhen, China). Eyes were coated with erythromycin ointment to avoid strong light exposure. The scalp was incised along the skull midline after sterilizing with iodophors. pAAV-CaMKIIα-ChR2(H134R)-mCherry (500 nL, 2–5 × 10^9^ pfu/mL; OBio Technology Shanghai, China) was injected into the vCA1 (AP –3.2 mm, ML +3.2 mm, DV –4.5 mm) at a rate of 50 nL/min. The needle syringe was kept for 10 min before withdrawal. The skin was sutured and then sterilized with iodophors. The mice were put on a heat lamp to revive. Three weeks after virus injection, optic fiber cannulas (NA = 0.37, Newdoon, China) were implanted into the vCA1 of mice (AP –3.2 mm, ML +3.2 mm, DV –4.1 mm). Four screws (RWD, Shenzhen, China) were anchored around the fiber cannulas with dental cement. Mice were put back into the cage for reviving postoperation.

### Optogenetic Stimulation and Seizures Induction

Mice were handled for consecutive 3 days before seizure induction and were temporarily anesthetized with isoflurane. The fiber cannulas were connected to a plug line with FC/PC joint at both ends. Seizures were induced 10 s after the mice woke up. Laser output is controlled by a function generator (Tektronix, China, NO. AFG 3022B). After many trials, the stimulation parameters were finally set as 20 Hz, blue light (472 nm), duty cycle 10%, pulse wave. The actual output power of optical fiber outlet was 2.8 mW/mm^2^ (when the waveform generator was not connected, the output power of optical fiber outlet was 43.2 mW/mm^2^). Mice behaviors were recorded using a digital camera. The severity of mice seizures was evaluated according to MRS (Racine, 1975; Haas et al., 1990): stage 1, facial convulsions, chewing behavior in the mouth; stage 2, chewing and rhythmic head nodding; stage 3, unilateral forelimb clonus; stage 4, bilateral forelimb clonus with rearing, standing on hind limb; stage 5, rearing and repeated falling; stage 6, wild running, jumping. Stages 1 to 3 were considered as focal seizures, and stages 4–6 considered generalized seizures. The time from stimulation onset to stage 4 was recorded as latency to generalized seizures in this study. There were some differences between Test 1 (Figures 1, 2) and Test 2 (Figure 4) in the duration of epileptic induction. The laser was turned off until stage 6 in Test 1, whereas stage 4 in Test 2. After the seizure induction, the mice were anesthetized by isoflurane, and the fiber was removed from their heads.

### Local Field Potentials Recording *in vivo*

Three weeks after virus injection into the vCA1, a 16-channel electrode was implanted into the ipsilateral M1 (+1.5 mm AP, +1.5 mm ML, –1.5 mm DV). The ground of the electrode was connected to two screws attached to the skull. Mice skin was sutured and then sterilized with iodophors. Mice were allowed 1 week for recovery postoperation before *in vivo* recording. Each mouse was handled for 5 min and adapted in a box for 10 min before the first time of recording. LFPs in M1 and mouse behaviors were recorded using a Recording System (Plexon, Hong Kong, China). Data were stored for offline analysis with 16-bit format, visualized in Neuro Explore. Amplitude and PSD of LFPs were analyzed in default parameters: shift (0.5 s), number of frequency values (8,192), normalization (log of PSD), show frequency from 0 to 150 HZ. Baseline signals were recorded before the laser was turned on. When the seizure induction began, mice behaviors were observed carefully, and the optogenetic stimulation was stopped at an appropriate time. After the final electrophysiological recording, the mice were killed, and the electrodes locations were confirmed by brain slice section.

### Open Field Test

The apparatus was a 60-cm × 60-cm × 50-cm plastic box. The floor was divided into nine equal squares, among which the whole central area takes up 50%. The individual mouse was placed in the center opposite to experimenter. Each mouse was allowed to explore freely for 5 min. Mice behaviors were recorded by a camera. The time and distance that the mice traveled in the central area were analyzed.

### Elevated-Plus Maze Test

The elevated plus maze consisted of two enclosed arms (30 cm × 5 cm × 20 cm) and two open arms (30 cm × 5 cm). The apparatus was elevated to 50 cm above the floor. Mice were placed individually in the center of the maze facing the open arm opposite to the experimenter. The time mice spent in the open/closed arms were recorded through tracking the center of the body. The mice explored freely for 5 min.

### Morris-Water Maze Test

The spatial learning and memory of mice were assessed by Morris-Water Maze test. For spatial learning, mice were trained in the maze to find a hidden platform for five consecutive days, three trials per day with an interval of 30 min from 2:00 to 5:00 PM each day. In each trial, mice started from one of the four quadrants facing the wall of the pool. Each trial ended when the animal climbed onto the platform. If the mice did not find the platform within 60 s, they were guided to the platform and allowed to stay on the platform for 15 s. The swimming path and time mice used to find the platform (escape latency) during training, as well as time mice pass through the previous platform quadrant in the test phase, were recorded by a video camera fixed to the ceiling, 1.5 m from the water surface.

### Western Blotting

Mice brains were removed, and the cortex and hippocampus were dissected on ice. Samples were homogenized with RIPA lysis buffer (Beyotime). Proteins were separated by sodium dodecyl sulfate–polyacrylamide gel electrophoresis, transferred onto nitrocellulose membranes (Merck Millipore) and then blocked with 5% bovine serum albumin (BSA). Blots were probed with polyclonal rabbit anti-Tau5 (1:1,000; cat no. MAB361, Millipore), polyclonal rabbit anti-AT8 (1:1,000; cat no. MN1020, Thermo), and polyclonal rabbit anti-α-tubulin (1:1,000; cat no. T9026, Sigma) and were then incubated with horseradish peroxidase–conjugated secondary antibodies and visualized by an enhanced chemiluminescence substrate system (Santa Cruz, CA, United States). Blots were visualized using an Odyssey Imaging System (LI-COR Biosciences) and quantitatively analyzed by ImageJ.

### Immunostaining

Mice were sacrificed 1 day after the last trial of WMW test and then anesthetized and perfused through ventriculus sinister with 0.9% NaCl for 5 min and then phosphate buffer containing 4% paraformaldehyde for 5 min. Brain were cryoprotected in 25% and 30% sucrose solutions in turn for 2 days. The next day, brains were cut into 30-μm sections using a cryostat microtome (CM1900, Leica). For immunohistochemistry, free-floating sections were blocked with 3% H_2_O_2_ in anhydrous methanol for 30 min, and non-specific sites were blocked with BSA for 30 min at room temperature. Sections were then incubated overnight at 4°C with Tau5 (1:200; cat no. MAB361, Millipore) or AT8 (1:200; cat no. MN1020, Thermo) antibodies. Immunoreactions were developed using a DAB-staining kit (ZSGB-BIO). For immunofluorescence, sections were thoroughly washed with PBS-T [phosphate-buffered saline (PBS) containing 0.1% Triton X-100] and incubated overnight with monoclonal mouse Tau 5 (1:200; cat no. MAB361, Millipore) or AT8 (1:200; cat no. MN1020, Thermo) antibodies. After that, the sections underwent PBS-T washes (three times, 5 min each), followed by 1-h incubation with the secondary antibody at 37°C. Finally, the slice underwent three more washes and counterstained with DAPI. Images were taken by two-photon laser-scanning confocal microscope (LSM710, Zeiss).

### Nissl Staining

Mice brain slices were selected and rinsed with PBS for 5 min and then moved to the adhesive slides with a brush and dried naturally. The tar-purple dye drops about 500 μL were spread evenly over the brain slices. Then, the slices dyed for about 10–15 min, distilled water for 1 min, 75% alcohol, 80% alcohol, 95% alcohol, and anhydrous ethanol for 1 min each, and finally made transparent with xylene for 30–60 min. Slices were sealed with neutral resin and dried naturally.

### Statistical Analyses

All data were analyzed and plotted using GraphPad Prism 7 (GraphPad Software, Inc., La Jolla, CA, United States). One-way analysis of variance (ANOVA), Tukey multiple-comparisons *post hoc* tests, and two-tailed unpaired *t* tests were used. *P* < 0.05 was considered significant. Data were shown as mean ± SEM.

## Data Availability Statement

The raw data supporting the conclusions of this article will be made available by the authors, without undue reservation.

## Ethics Statement

The animal study was reviewed and approved by institutional guidelines and the Animal Care and Use Committee of Huazhong University of Science and Technology.

## Author Contributions

YG, JZ, and J-ZW conceived the concept, instructed data analysis, and revised the manuscript. YG and JZ conducted most of the data analysis, prepared the figures, and wrote the manuscript draft. TJ, GP, FS, and RX helped with some experiments. JW reviewed the manuscript with input from all authors. All authors contributed to the article and approved the submitted version.

## Conflict of Interest

The authors declare that the research was conducted in the absence of any commercial or financial relationships that could be construed as a potential conflict of interest.
